# Difference in the integrated effects of sympathetic vasoconstriction and local vasodilation in human skeletal muscle and skin microvasculature

**DOI:** 10.14814/phy2.14070

**Published:** 2019-04-13

**Authors:** Masashi Ichinose, Mikie Nakabayashi, Yumie Ono

**Affiliations:** ^1^ Human Integrative Physiology Laboratory School of Business Administration Meiji University Tokyo Japan; ^2^ Graduate School of Science and Technology Meiji University Kanagawa Japan; ^3^ Department of Electronics and Bioinformatics School of Science and Technology Meiji University Kanagawa Japan

**Keywords:** Diffuse correlation spectroscopy, integrated circulatory regulation, metabolic vasodilation, neural cardiovascular regulation, reactive hyperemia

## Abstract

We investigated the integration of sympathetic vasoconstriction and local vasodilation in the skeletal muscle and skin microvasculature of humans. In 39 healthy volunteers, we simultaneously measured the blood flow index in the flexor carpi radialis muscle using diffuse correlation spectroscopy and the skin using laser‐Doppler flowmetry. We examined the effects of acute sympathoexcitation induced by forehead cooling on relatively weak and robust vasodilatory responses during postocclusive reactive hyperemia (PORH) induced by 70‐sec and 10‐min arterial occlusion in the upper arm. To increase sympathetic tone during PORH, forehead cooling was begun 60 sec before the occlusion release and ended 60 sec after the release. In the 70‐sec occlusion trials, acute sympathoexcitation reduced the peak and duration of vasodilation in both skeletal muscle and skin. The inhibition of vasodilation by sympathoexcitation was blunted in both tissues by the robust vasodilatory stimulation produced by the 10‐min occlusion, and the degree of blunting was greater in skeletal muscle than in skin, especially the initial and peak responses. Sympathoexcitation reduced the peak vasodilation only in skin, while it accelerated the initial vasodilation only in skeletal muscle. However, the decline in vasodilation after the peak was significantly hastened in skeletal muscle, shortening the duration of the vasodilation. We conclude that, in humans, the integration of sympathetic vasoconstriction and local vasodilation has different effects in skeletal muscle and skin and is likely an important contributor to the selective control of perfusion in the microcirculations of different tissues.

## Introduction

A rapid marked increase in limb blood flow is observed following ischemia caused by temporary arterial occlusion. This phenomenon, postocclusive reactive hyperemia (PORH), reflects myogenic and local metabolic and/or endothelial factors within the resistance vasculature and thus can be used as a test of microvascular function (Blair et al. 1959; Kidawa et al. 2003; Nakamura et al. 2005; Frisbee 2006; Ono et al. 2018). Elevated adrenergic vascular tone is one of the factors that compromises the capacity for vasodilation in such pathological states as diabetes mellitus and metabolic syndrome (Friedman 1989; Frisbee 2004, 2006; Ono et al. 2018). It has been shown, for example, that sympathetic vascular tone in humans restrains PORH assessed in the forearm as a whole (Sinoway et al. 1988; Moradkhan et al. 2007). But those results reflect the responses of all the tissues comprising the forearm, including skeletal muscles, skin, adipose and bone. In a recent study, we focused on the integration of sympathetic vasoconstriction and local vasodilation specifically within the skeletal muscle microvasculature in humans (Ichinose et al. 2018). Our use of near‐infrared diffuse correlation spectroscopy (DCS) enabled us to demonstrate that acute sympathoexcitation attenuates PORH in the human skeletal muscle microcirculation (Ichinose et al. 2018). In contrast to conventional PORH in the whole forearm, direct examination of skeletal muscle microvascular function using DCS has potential clinical implications, as skeletal muscle is the major site of glucose uptake and storage (Baron et al. 1988), and pathological degeneration of microvascular function and the resultant perfusion insufficiency could potentially contribute to systemic insulin resistance (Lillioja et al. 1987; Clark 2008; Groen et al. 2014). In addition, impaired skeletal muscle perfusion would also lead to exercise intolerance (Rowell 1993; Frisbee 2004; Kaur et al. 2018), and the resultant inactivity would likely worsen disease conditions.

To regulate skeletal muscle blood flow in response to changes in metabolic demand, it may be effective to blunt sympathetic vasoconstriction through enhanced metabolic vasodilatory stimuli (Thomas et al. 1994; Tschakovsky et al. 2002; Calbet and Joyner 2010). Indeed, local vasodilatory mechanisms have been shown to impair sympathetic constrictor effectiveness within contracting skeletal muscle through direct action on vascular smooth muscle (Thomas et al. 1994; Hearon et al. 2017). This effect, termed functional sympatholysis, ensures adequate blood flow to active skeletal muscle, despite elevated sympathetic vasoconstrictor activity (Thomas et al. 1994; Tschakovsky et al. 2002; Hearon et al. 2017). However, it is unknown whether the sympathetically mediated inhibition of dilation of skeletal muscle microvessels during PORH is affected by the levels of local vasodilatory stimuli. In addition, because tissues and organs vary widely in their metabolic activities and blood flow, the integrated effects of sympathetic vasoconstriction and local vasodilation may differ in the microcirculations of different tissues in humans. In that case, heightened sympathetic tone in pathological states may differentially diminish microvascular function in different tissues, this issue, however, has also not been resolved.

Skeletal muscle and skin are both under strong sympathetic vasomotor control, but the mechanisms governing blood flow in these tissues largely differ, reflecting their different physiological functions (Rowell 1993; Minson et al. 2001; Hodges et al. 2009; Kenney et al. 2014). Control of skeletal muscle blood flow is primarily to regulate O_2_ delivery to meet metabolic demand, which usually exceeds the capacity for O_2_ delivery during dynamic exercise at higher than moderate intensities (Rowell 1993; Calbet and Joyner 2010; Ichinose et al. 2015). On the other hand, blood flow through the skin is important for thermoregulation, and the capacity of cutaneous vasodilation and elevation of blood flow for that purpose is greatly in excess of the metabolic needs of the skin (Rowell 1993; Minson et al. 2001; Kenney et al. 2014). During exercise under heat stress, active skeletal muscle competes with skin for blood flow, which impairs both thermoregulation and exercise performance (Gonzalez‐Alonso et al. 2008; Kenney et al. 2014). Furthermore, because peripheral blood flow capacity exceeds the pumping capacity of a healthy human heart, preventing excessive vasodilation in both skeletal muscle and skin is essential for maintaining arterial blood pressure (Andersen and Saltin 1985; Sheriff et al. 1993; Calbet et al. 2004; Gonzalez‐Alonso et al. 2008; Calbet and Joyner 2010; Kenney et al. 2014). Consequently, the integrated effects of local vasodilation and sympathetic vasoconstriction in skeletal muscle and skin may be deeply involved in determining the distribution of blood flow to these tissues and also in systemic arterial blood pressure regulation. This makes skeletal muscle and skin suitable representative tissues for investigating differences in the integration of sympathetic vasoconstriction and local vasodilation during PORH.

In this study, therefore, we investigated the effects of increased sympathetic tone on vasodilatory responses within the forearm skeletal muscle and skin microvasculature during PORH. For this purpose, we used DCS and laser‐Doppler flowmetry to simultaneously measure blood flow in the skeletal muscle and skin microcirculations. It has been shown in the forearm that PORH increases with prolongation of ischemia. However, if the period of ischemia exceeds about 3 min, peak vasodilation reaches a maximum, and further increases in PORH mainly reflect increases in the duration of the vasodilation (Carlsson et al. 1987). Based on these earlier findings, occlusion for 70 sec has been used to produce relatively weak vasodilatory stimulation, which would not induce maximum peak forearm vasodilation during PORH. On the other hand, robust vasodilatory stimulation was induced by occlusion for 10 min, which causes maximum peak vasodilation and substantially prolongs the duration of forearm vasodilation during PORH (Carlsson et al. 1987; Sinoway et al. 1988). Acute sympathoexcitation can be induced during PORH through forehead cooling (Sinoway et al. 1988; Heindl et al. 2004; Muller et al. 2014). Using this experimental approach, we investigated the effects of elevated sympathetic tone on both maximal vasodilatory capacity and mild submaximal vasodilation, which may represent the more usual vasomotion. We anticipated that examining such a wide range of vasodilatory responses would provide insight into microvascular regulation in various situations associated with heightened sympathetic tone, such as during exercise, hyperthermia and hypoxemia, and in pathological conditions. We hypothesized (1) that acute sympathoexcitation would diminish local vasodilation during PORH in both the skeletal muscle and skin microvasculature; (2) that this sympathetically mediated inhibition of vasodilation would be blunted by robust vasodilatory stimulation; and (3) that the blunting of sympathetic inhibition of vasodilation would be greater in the skeletal muscle than skin microcirculation.

## Methods

### Subjects

We studied 39 healthy volunteers (37 men and 2 women) with a mean age of 20 ± 0.2 year, body weight of 68.5 ± 2.1 kg, and height of 172.1 ± 1.0 cm. None of the subjects were receiving medication and none smoked. Subjects were instructed to abstain from caffeine, exercise, and alcohol beginning 12 h prior to the experiment. Based on self‐reports, both female subjects were studied during the early follicular phase of their menstrual cycle. The study was carried out in accordance with the Declaration of Helsinki and was approved by the institutional review board of the School of Science and Technology of Meiji University. Each subject gave informed written consent.

### Procedures

The experiments were conducted in a room maintained at 25°C. After entering the test room, each subject adopted a supine position, and a rapidly inflatable cuff for arterial occlusion was placed on the right upper arm. After setting up the equipment, there was a rest period of at least 15 min before data collection was begun.

The effects of sympathoexcitation on hemodynamic responses during PORH were examined as follows. After baseline resting measurements were recorded for 2 min, the occlusion cuff on the subject's right upper arm was inflated to supersystolic pressure (>250 mmHg). The cuff pressure rose above systolic blood pressure in less than 1 sec. This quick inflation of the cuff prevents significant venous pooling. The cuff remained inflated for 70 sec or 10 min, after which PORH was induced by releasing the arterial occlusion. To increase sympathetic tone, we applied a bag of ice firmly to the subject's forehead (Sinoway et al. 1988; Heindl et al. 2004; Muller et al. 2014). The forehead cooling was initiated 60 sec before release of the arterial occlusion and stopped 60 sec after the release. This protocol was chosen because we found in a previous study that vasoconstriction reached a maximum and the rise in mean arterial pressure (MAP) reached a near‐steady state approximately 60 sec after the start of forehead cooling (Ichinose et al. 2018). Data recording was continued for 5 min after the release of the occlusion. In each subject, inductions of PORH after 70‐sec and 10‐min occlusions with and without forehead cooling were conducted in random order. After each trial, there was a rest period of at least 15 min. Before beginning the next trial, we monitored the hemodynamic parameters and confirmed the values were at the resting levels. To prevent extraneous light from contaminating the DCS signal to the detector probe, experiments were performed in a dark room. All the experiments were completed within 1 day.

### Measurements

Heart rate (HR) was monitored using a three‐lead electrocardiogram (ECG). Beat‐to‐beat changes in arterial blood pressure were assessed using finger photoplethysmography (Finometer; Finapres Medical Systems, Netherlands); the monitoring cuff was placed around the middle finger of the left hand, with the forearm and hand supported so that the cuff was aligned at the level of the heart. The pressure in the occlusion cuff was measured using a pressure transducer.

DCS is an emerging optical technique for noninvasive measurement of deep tissue hemodynamics (Durduran et al. 2010). A DCS system constructed in our laboratory was used to evaluate hemodynamics in the skeletal muscle microcirculation. A detailed description of the DCS system is provided elsewhere (Nakabayashi and Ono 2017; Ono et al. 2018). Briefly, the system consists of a long coherence, continuous wave laser operating at 785 nm (DL 785‐100‐S, 100 mW; CrystaLaser, USA), which serves as the source, and a photon‐counting avalanche‐photodiode, which serves as the detector (COUNT‐T‐100‐FC; Laser Components, Germany). The correlation equations and algorithms used for the DCS are described in detail by Durduran et al. (2010) and Dong et al. (2012). The DCS system measures intensity fluctuations in a reflected near‐infrared light signal caused primarily by moving red blood cells and provides a microvascular blood flow index (BFI). An in‐house program developed with the aid of LabVIEW (version 2016; National Instruments, USA) was used to collect the light intensity data at a sampling rate of 100 kHz and determine the BFI every 1 sec. Photo‐emitter and detector probes were placed on the skin surface above the flexor carpi radialis muscle in the right forearm. The interprobe distance was 3 cm. The probe pair was housed in a rubber holder, which ensured that the position of the probes relative to each other was invariant. The probes within the rubber holder were secured on the skin surface using surgical tape. The approximate depth of the DCS measurement below the skin surface was estimated to be half the inter‐probe distance (~1.5 cm) (Yu et al. 2005). DCS has been validated in both the skeletal muscle and cerebral microcirculation against several standards, including arterial spin‐labeled (Yu et al. 2007) and phase contrast MRI (Jain et al. 2014), fluorescent microspheres (Zhou et al. 2009), Doppler ultrasound (Roche‐Labarbe et al. 2010), and time‐resolved NIRS with administration of a flow tracer (Diop et al. 2011).

Skin blood flow was measured using laser‐Doppler flowmetry (ALF21, Advance, Japan) and expressed as laser‐Doppler flow (LDF). The LDF probe was positioned on the ventral forearm 5 cm distal from the DCS detector probe (i.e., 8 cm distal from the emitter probe). With that distance between the probes, the on and off of the DCS and LDF lights did not affect their respective measurements, confirming that the lights from the DCS and LDF did not interfere with each other. LDF measurements are specific to the skin and are not affected by blood flow to the underlying skeletal muscle (Saumet et al. 1988).

The analog signals representing the ECG, blood pressure waveform, LDF and occlusion cuff pressure were digitized at a sampling frequency of 1 kHz through an analog‐to‐digital converter (NI DAQCard‐6062E, National Instruments, USA) and fed into a personal computer. Beat‐to‐beat HR and MAP were calculated using an off‐line data‐analysis program and then interpolated and resampled at 1 Hz to obtain data at the same time intervals as the BFI. The LDF data were averaged every second. To quantify relative changes in blood flow within the skeletal muscle and skin microcirculations, BFI and LDF values were normalized to the mean values during the rest period, as in previous studies (Yu et al. 2005, 2007; Yamazaki et al. 2006; Nakabayashi and Ono 2017; Ichinose et al. 2018). BFI/MAP was calculated as an index of skeletal muscle vascular conductance (MVC). Cutaneous vascular conductance (CVC) was calculated as LDF/MAP. MVC and CVC values were also normalized to the resting values, as with BFI and LDF.

In a separate resting session, the thickness of the near‐surface layers (skin and adipose tissue) above the muscle at the sites where the DCS probes were fixed was measured in all subjects using an ultrasound system (iU22; Philips, Netherlands) operated in B‐mode with a 17 MHz linear array transducer probe (model L17‐5). The thickness of the near‐surface layers at the DCS measurement sites was 3.0 ± 0.2 mm.

### Data analysis

Hemodynamic values at rest were calculated by averaging the data obtained during 2 min of the resting period. The BFI, LDF, MVC, and CVC responses during PORH were evaluated by quantifying the following parameters: (1) peak values, (2) time to reach the peak after cuff release, (3) time from the cuff release until the responses decayed to 50% of the peak, and (4) total perfusion and vasodilatory responses assessed as the area under each measurement during the first 15 sec, 1 min, and 5 min of PORH. To examine the degree of the PORH inhibition induced by sympathoexcitation, the percentage change in the peaks and total responses from the control to the forehead cooling conditions were calculated for the 70 sec and 10 min occlusion trials. In addition, the difference in the percentage change of each parameter between the two occlusion trials was calculated as values from the 10 min occlusion trials minus those from the 70 sec occlusion trials. This was done to compare the effect of lengthening of occlusion period on the sympathetically mediated inhibition of PORH in the skeletal muscle and skin microvasculatures. BFIs with serious artifacts characterized by brief periods of spiking evoked by apparent body motion were excluded from the analysis. Due to technical difficulties, we were unable to measure LDF in three male subjects. Therefore, 36 data sets (34 men and 2 women) were analyzed for LDF and CVC.

### Statistical analysis

Data are presented as the mean ± SE. Two‐way repeated measures analysis of variance followed by Fisher's post hoc comparisons test was used to compare the cardiovascular responses at rest and during forehead cooling between the control and forehead cooling conditions in the 70 sec and 10 min occlusion trials. One‐way repeated‐measures analysis of variance was used to compare the values of parameters used to quantify PORH between the control and forehead cooling conditions in the two occlusion periods. The percentage changes in each parameter from the control to the forehead cooling conditions in the 70‐sec and 10‐min occlusion trials were compared using Student's paired *t* test, which was also used to compare the difference in the percentage changes of each parameter between the two occlusion trials in skeletal muscle and skin. Because of the lack of LDF measurements for three subjects, comparisons of PORH responses between the BFI and LDF and between the MVC and CVC were made using the 36 available data sets. These were also done using Student's paired *t* test. Values of *P* < 0.05 were considered significant.

## Results

### Effect of forehead cooling on MAP and HR

**Figure 1:**
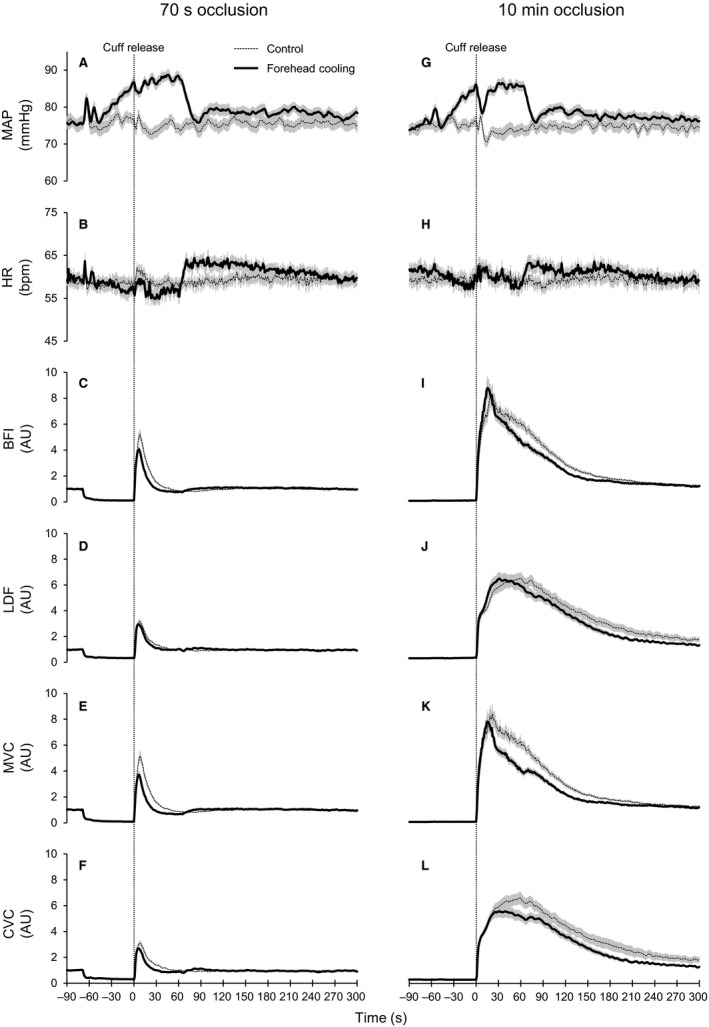
Ensemble‐averaged hemodynamic responses derived during PORH in the control and forehead cooling conditions. (A–L) responses observed in the 70‐sec (A–F) and 10‐min (G–L) occlusion trials. Dotted and solid lines show ensemble‐averaged values in the control and forehead cooling conditions, respectively. Gray bars indicate SE. Dashed lines at 0 sec indicate the time of occlusion cuff release. Forehead cooling started 60 sec before the cuff release (−60 sec) and ended 60 sec after cuff release (60 sec). Abbreviations: MAP, mean arterial pressure; HR, heart rate [in beats/min (bpm)]; BFI, blood flow index of skeletal muscle derived by DCS; LDF, skin blood flow derived by laser‐Doppler flowmetry; MVC, skeletal muscle vascular conductance; CVC, cutaneous vascular conductance. AU, arbitrary units (i.e., values normalized against the resting levels). *n* = 39 for MAP, HR, BFI and MVC and 36 for LDF and CVC.

**Figure 2:**
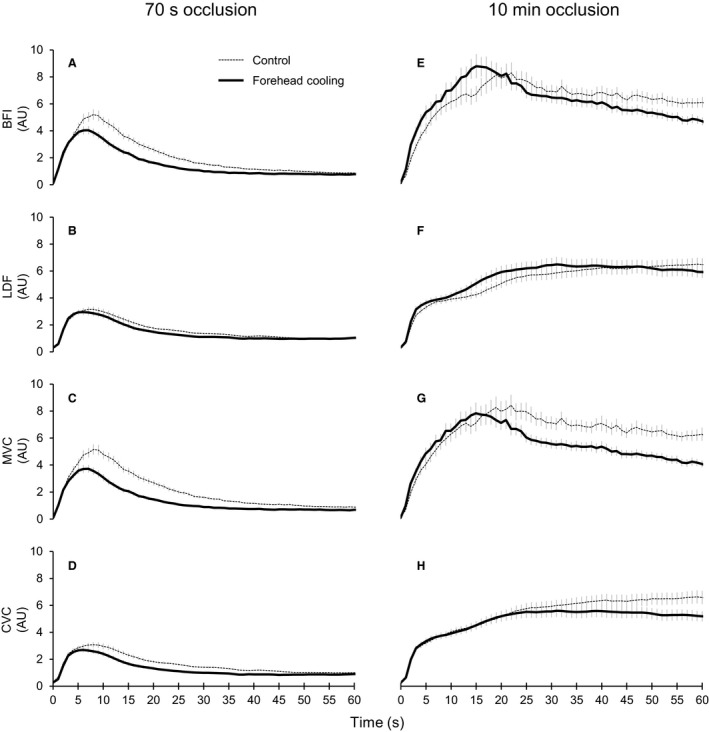
Ensemble‐averaged blood flow and vascular conductance responses during the first minute of PORH. (A–H) responses observed in the 70‐sec (A–D) and 10‐min (E–H) occlusion trials. Dotted and solid lines show ensemble‐averaged values in the control and forehead cooling conditions, respectively. Gray bars indicate the SE. The occlusion cuff was released at 0 sec. Abbreviations: BFI, blood flow index of skeletal muscle derived by DCS; LDF, skin blood flow derived by laser‐Doppler flowmetry; MVC, skeletal muscle vascular conductance; CVC, cutaneous vascular conductance. AU, arbitrary units (i.e., values normalized against the resting levels). *n* = 39 for BFI and MVC and 36 for LDF and CVC.

Figure 1 shows ensemble‐averaged hemodynamic responses during PORH under the control and forehead cooling conditions in the 70‐sec and 10‐min occlusion trials. To focus on the initial hyperemia and vasodilatory responses, the ensemble‐averaged blood flow and vascular conductance responses during the first minute of PORH are shown in Figure 2. Table 1 shows MAP and HR at rest and during the second minute of forehead cooling. The resting MAP and HR levels did not differ between conditions or between occlusion periods. After the start of forehead cooling, MAP gradually increased until it reached a near‐steady state at approximately 60 sec (Fig. 1A and G). The mean MAP during the 60‐sec period after cuff release was significantly higher during forehead cooling than under control conditions in both occlusion periods. The release of the 10‐min occlusion produced an abrupt, transient decrease in MAP (Fig. 1G), which is likely due to robust vasodilation within the forearm. Consequently, the mean MAP was slightly but significantly lower than the resting value in the control condition. However, this effect was not large enough to produce a statistically significant difference in mean MAP between the 70‐sec and 10‐min occlusion trials. Similarly, the mean MAP during forehead cooling did not differ between the two occlusion periods. Forehead cooling slightly but significantly decreased HR from the resting level in the 70‐sec occlusion trial, but the responses were not large enough to produce a statistically significant difference between the conditions or the occlusion periods.

**Table 1:** MAP and HR during the rest and forehead cooling periods in the 70‐sec and 10‐min occlusion trials

		Rest	Second minute of forehead cooling
MAP (mmHg)	Con70s	76 ± 1.5	75 ± 1.5
	FC70s	77 ± 1.3	87 ± 1.5*,†
	Con10m	75 ± 1.3	73 ± 1.3*
	FC10m	75 ± 1.2	84 ± 1.5*,†
HR (beats/min)	Con70s	59 ± 1.4	59 ± 1.3
	FC70s	60 ± 1.3	57 ± 1.6*
	Con10m	60 ± 1.7	60 ± 1.3
	FC10m	59 ± 1.5	60 ± 1.6

Values are means ± SE. Abbreviations: MAP, mean arterial pressure; HR, heart rate; Con70s and FC70s, control and forehead cooling conditions in the 70‐sec occlusion trials, respectively; Con10m and FC10m, control and forehead cooling conditions in the 10‐min occlusion trials, respectively. *P < 0.05, rest versus forehead cooling, ^†^ *P* < 0.05, control versus forehead cooling conditions in the same occlusion periods.

### Effect of forehead cooling on PORH in skeletal muscle and skin

**Figure 3:**
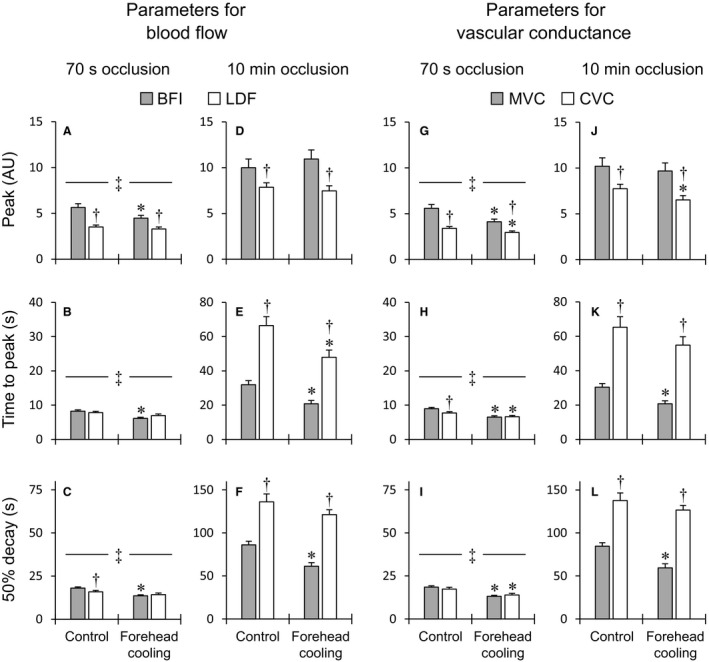
Group‐averaged values of the peak, time to peak and 50% decay time for blood flow (A–F) and vascular conductance (G–L) responses during PORH. Abbreviations: BFI, blood flow index of skeletal muscle derived by DCS; LDF, skin blood flow derived by laser‐Doppler flowmetry; MVC, skeletal muscle vascular conductance; CVC, cutaneous vascular conductance. AU, arbitrary units (i.e., values normalized against the resting levels). ^‡^ *P* < 0.05, 70‐sec versus 10‐min occlusion trials under the same conditions in skeletal muscle and skin. *P < 0.05, control versus forehead cooling in same occlusion period, ^†^ *P* < 0.05, BFI versus LDF or MVC versus CVC under the same conditions. *n* = 39 for BFI and MVC and 36 for LDF and CVC.

Group‐averaged values for the peak, time to peak, and 50% PORH decay time are shown in Figure 3. The release of occlusion evoked a clear transient overshoot in blood flow and vascular conductance within both the skeletal muscle and skin in both occlusion trials (Figs. 1C‐F, I‐L and 2). As expected, the 10‐min occlusion produced markedly larger PORH responses than the 70‐sec occlusion. Indeed, all the parameters quantifying PORH in both tissues were significantly greater after 10 min than 70 sec, regardless of the conditions (*P* < 0.05). The forehead cooling in the 70‐sec occlusion trials significantly reduced the peak BFI, MVC, and CVC compared to control, while the peak LDF did not differ (Fig. 3A, G). In the 10‐min occlusion trial, however, forehead cooling only decreased the peak CVC; peak MVC did not change (Fig. 3D and J). In this situation, peak BFI tended to increase as compared to control (*P* = 0.078), though the effect was not statistically significant (Figs. 2E and 3D). Peak LDF did not change. The times to peak BFI, MVC, and CVC were shorter than control during forehead cooling in the 70‐sec occlusion trial (Fig. 3B and H). The time to peak LDF did not significantly change. In the 10‐min occlusion trial, the times to peak BFI, LDF, and MVC were significantly shortened during forehead cooling (Fig. 3E and K). The time to peak CVC also tended to be shortened during forehead cooling (*P* = 0.053). In the 70‐sec occlusion trials, the 50% decay times for BFI, MVC, and CVC were shorter during forehead cooling than in the control condition, while the 50% decay time for LDF did not significantly differ between conditions (Fig. 3C and I). In the 10‐min occlusion trial, forehead cooling shortened the 50% decay times for BFI and MVC but had only a limited effect on the decay times for LDF and CVC (Fig. 3F and L).

**Figure 4:**
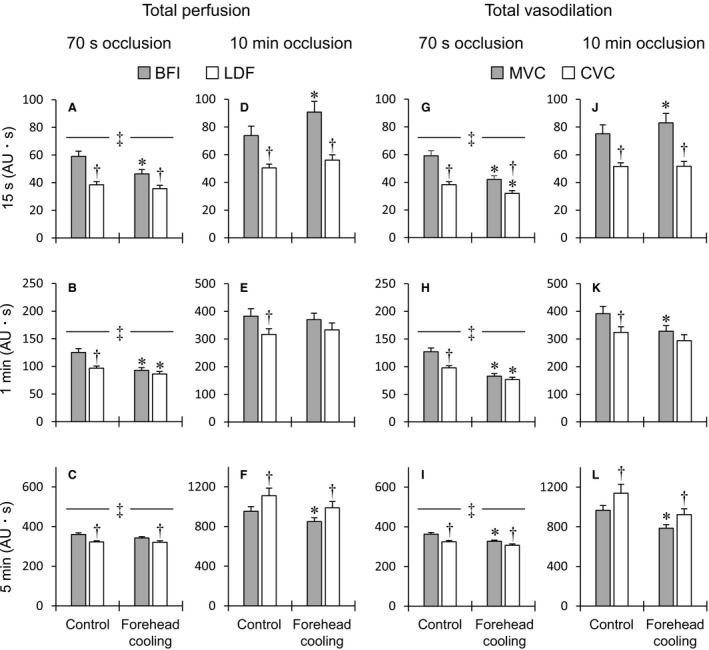
Group‐averaged values of the total perfusion (A–F) and vasodilation (G–L) responses during the first 15 sec, 1 min, and 5 min of PORH. Abbreviations: BFI, blood flow index of skeletal muscle derived by DCS; LDF, skin blood flow derived by laser‐Doppler flowmetry; MVC, skeletal muscle vascular conductance; CVC, cutaneous vascular conductance. AU, arbitrary units (i.e., values normalized against the resting levels). ^‡^ *P* < 0.05, 70‐sec versus 10‐min occlusion trials under the same conditions in skeletal muscle and skin. *P < 0.05, control versus forehead cooling in the same occlusion period, ^†^ *P* < 0.05, BFI versus LDF or MVC versus CVC under the same conditions. *n* = 39 for BFI and MVC and 36 for LDF and CVC.

The total perfusion and vasodilatory responses during the first 15 sec, 1 min, and 5 min of PORH are presented in Figure 4. In the 70‐sec occlusion trials, sympathoexcitation elicited by forehead cooling apparently inhibited the initial vasodilatory responses in both skeletal muscle and skin within the first 15 sec (Fig. 2C and D). Consequently, the total BFI, MVC, and CVC responses during the first 15 sec were significantly reduced (Fig. 4A and G). The total LDF response during the first 15 sec did not change. By contrast, in the 10‐min occlusion trials, sympathoexcitation accelerated the initial vasodilation and perfusion only in the skeletal muscle microcirculation (Fig. 2E–H). As a result, the total BFI and MVC responses during the first 15 sec were significantly greater during forehead cooling (Fig. 4D and J). On the other hand, forehead cooling had no significant effect on the total LDF and CVC responses during the first 15 sec. In the 70‐sec occlusion trials, the total perfusion and vasodilation during the first 1 min in both the skeletal muscle and skin microcirculation significantly decreased during forehead cooling (Fig. 4B and H). Forehead cooling in the 10‐min occlusion trials significantly reduced the total response during the first 1 min for MVC, but had limited effects on the total BFI, LDF, and CVC responses (Fig. 4E and K). Forehead cooling in the 70‐sec occlusion trials also significantly decreased the total MVC response during the first 5 min of PORH and also tended to decrease the total CVC response (*P* = 0.057) (Fig. 4I). Forehead cooling did not significantly affect the total BFI and LDF responses during the first 5 min (Fig. 4C). In the 10‐min occlusion trials, forehead cooling significantly decreased the total BFI and MVC responses during the first 5 min and also tended to reduce the total CVC response (*P* = 0.054), but it had a limited effect on the LDF response (Fig. 4F and L).

### Comparison of PORH in skeletal muscle and skin

When comparing PORH in the skeletal muscle and skin microcirculations, peak blood flow and vascular conductance were significantly higher in skeletal muscle than in skin, regardless of the occlusion periods or conditions (Fig. 3A, D, G and J). In the 70‐sec occlusion trials, the times to peak CVC were shorter than to peak MVC under control conditions, but they were decreased by forehead cooling, and the two conductances became similar (Fig. 3H). The times to peak BFI and LDF were comparable in both conditions (Fig. 3B). However, in the 10‐min occlusion trials, the times to peak blood flow and vascular conductance in the skin were substantially greater than in skeletal muscle (Fig. 3E and K). In the 70‐sec occlusion trials, the 50% decay time for LDF was slightly but significantly shorter than for BFI in the control condition, but the decay times for LDF and BFI were similar in the forehead cooling conditions (Fig. 3C). The 50% decay times for MVC and CVC were comparable in both conditions (Fig. 3I). In the 10‐min occlusion trials, however, the 50% decay times for blood flow and vascular conductance in skin were markedly longer than in skeletal muscle, regardless of the condition (Fig. 3F and L). The total perfusion and vasodilation during the first 15 sec in skeletal muscle were greater than in skin, regardless of the occlusion period or condition (Fig. 4A, D, G and J). In both occlusion trials, the total perfusion and vasodilation during the first 1 min were greater in skeletal muscle than in skin in the control condition, but in the forehead cooling condition they did not significantly differ (Fig. 4B, E, H and K). In the 70‐sec occlusion trials, the total perfusion and vasodilation during the first 5 min were greater in skeletal muscle than in skin in both conditions (Fig. 4C and I). In the 10‐min occlusion trials, by contrast, total perfusion and vasodilation were greater during the first 5 min in skin than in skeletal muscle in both conditions (Fig. 4F and L).

### The blunting of sympathetic inhibition of PORH by lengthening of ischemia

Table 2 shows the percentage changes in peak and total perfusion or vasodilation during PORH from the control to the forehead cooling conditions and the differences in the percentage changes between the two occlusion trials. The percentage changes in peak BFI and MVC elicited by forehead cooling were significantly larger in the 10‐min than the 70‐sec occlusion trials, whereas the percentage changes in peaks LDF and CVC were similar in the two trials. This indicates that the sympathetically mediated inhibition of peak hyperemia and vasodilation was blunted by the lengthening of the occlusion period in skeletal muscle but not in skin. The percentage changes in total perfusion and vasodilation during the first 15 sec and 1 min in both skeletal muscle and skin were significantly greater in the 10‐min than 70‐sec occlusion trials. Lengthening the ischemia thus blunted sympathetic inhibition of PORH, as total responses during the first 15 sec and 1 min were affected in both the skeletal muscle and skin microcirculation. In both skeletal muscle and skin, the percentage changes in total perfusion and vasodilation during the first 5 min of PORH did not significantly differ between occlusion trials. Forehead cooling in the 70‐sec trials tended to induce greater reductions in total perfusion and vasodilation during the first 1 min in skeletal muscle than in skin (BFI vs. LDF, *P* = 0.052; MVC vs. CVC, *P* = 0.063). On the contrary, in the 10‐min occlusion trials, the percentage changes in total perfusion and vasodilation during the first 15 sec tended to be larger in skeletal muscle than in skin (BFI vs. LDF, *P* = 0.072; MVC vs. CVC, *P* = 0.068). These results reflect the significant acceleration of initial hyperemia and vasodilation responses observed selectively in the skeletal muscle during sympathoexcitation in the 10‐min occlusion trials (Figs. 2E–H and 4D and J). In addition, the differences in the percentage changes in the peak and total perfusion and vasodilation during the first 15 sec in skeletal muscle were significantly greater than in skin. These findings show that the sympathetic inhibition of the peak and initial responses during PORH are blunted to a greater degree by lengthening the ischemia in the skeletal muscle than the skin microcirculation. No significant differences were observed in the differences in the percentage changes in other parameters between skeletal muscle and skin.

**Table 2:** Percentage changes in peak and total perfusion or vasodilation during PORH from the control to the forehead cooling condition in the 70‐sec and 10‐min occlusion trials and the differences in the percentage change between the two occlusion trials

		70‐sec occlusion (%)	10‐min occlusion (%)	Differences (∆%; 10 min–70 sec)
Peak	BFI	−12 ± 6	14 ± 6*	25 ± 7
	LDF	−3 ± 5	1 ± 7	4 ± 7 ^†^
	MVC	−19 ± 5	−2 ± 5*	15 ± 6
	CVC	−10 ± 4	−10 ± 6	0 ± 6 ^†^
Total perfusion or vasodilation for 15 sec	BFI	−14 ± 6	30 ± 7*	43 ± 9
	LDF	−5 ± 4	15 ± 8*	21 ± 8 ^†^
	MVC	−22 ± 5	15 ± 6*	37 ± 8
	CVC	−14 ± 4	3 ± 6*	17 ± 6 ^†^
Total perfusion or vasodilation for 1 min	BFI	−21 ± 5	3 ± 5*	21 ± 6
	LDF	−10 ± 4	12 ± 8*	22 ± 7
	MVC	−30 ± 4	−11 ± 5*	17 ± 6
	CVC	−21 ± 3	−4 ± 6*	17 ± 6
Total perfusion or vasodilation for 5 min	BFI	−3 ± 3	−6 ± 5	−4 ± 5
	LDF	1 ± 3	2 ± 8	1 ± 8
	MVC	−8 ± 3	−13 ± 5	−7 ± 5
	CVC	−5 ± 3	−6 ± 7	−1 ± 7

Values are means ± SE. Abbreviations: BFI, blood flow index of skeletal muscle derived by DCS; LDF, skin blood flow derived by laser‐Doppler flowmetry; MVC, skeletal muscle vascular conductance; CVC, cutaneous vascular conductance. Differences were calculated as values in the 10‐min trials minus those in the 70‐sec trials. Greater differences indicate greater blunting of the sympathetic inhibition of PORH by the lengthening of ischemia. *P < 0.05, 70‐sec versus 10‐min occlusion trials, ^†^ *P* < 0.05, BFI versus LDF or MVC versus CVC.

## Discussion

To the best of our knowledge, this is the first study examining the effect of acute sympathoexcitation on local vasodilation in both the skeletal muscle and skin microvasculature in humans. The major findings of this investigation are as follows. (1) Acute sympathoexcitation induced by forehead cooling inhibits local vasodilation in both the skeletal muscle and skin microvasculature during PORH. (2) This sympathetic inhibition of vasodilation is blunted when local vasodilation is enhanced by lengthening the ischemia. (3) This blunting of sympathetic inhibition is greater in the skeletal muscle than skin microcirculation, especially the initial and peak hyperemia and vasodilatory responses. (4) Under robust local vasodilatory stimulation, sympathoexcitation accelerated the initial vasodilation and hyperemia selectively in the skeletal muscle microvasculature.

PORH occurs as a result of local vasodilatory mechanisms elicited by transient arterial occlusion (Blair et al. 1959; Carlsson et al. 1987; Crecelius et al. 2013). Local metabolic or endothelial factors within the resistance vasculature, including prostaglandins (Carlsson et al. 1987; Engelke et al. 1996), adenosine (Carlsson et al. 1987), and nitric oxide (Tagawa et al. 1994; Engelke et al. 1996), are thought to be involved. In addition, the myogenic vasodilation caused by decreases in vascular myogenic tone during occlusion also appears to contribute to the hyperemia, particularly at early times (Carlsson et al. 1987; Engelke et al. 1996; Koller and Bagi 2002). According to Crecelius et al. (Crecelius et al. 2013), combined inhibition of inwardly rectifying potassium channels (K_IR_) and Na+/K+‐ATPase nearly abolished forearm PORH in humans. Thus, regardless of the initiating event, the relaxation of vascular smooth muscle within the resistance vessels appears to be largely due to signaling pathways mediated through activation of K_IR_ and Na+/K+‐ATPase. Moreover, sympathetic vascular tone has been shown to modulate PORH assessed in the forearm as a whole (Sinoway et al. 1988; Moradkhan et al. 2007). In this study, we provide further insight into the integration of sympathetic vasoconstriction and local vasodilation during PORH in the human skeletal muscle and skin microvasculature. When relatively weak local vasodilatory stimuli were produced by occlusion for 70 sec, acute sympathoexcitation started to reduce vasodilation within the initial 15 sec of the PORH in both skeletal muscle and skin. Consequently, both the peak and duration of the vasodilation decreased, leading to significant decreases in total vasodilation. By contrast, when robust vasodilatory stimulation was induced by occlusion for 10 min, the sympathetic inhibitory effects on vasodilation during the initial approximately 15 sec of PORH completely disappeared. Instead, the acute sympathoexcitation selectively accelerated the initial hyperemia and vasodilation in the skeletal muscle microvasculature, as demonstrated by the increased total perfusion and vasodilation during the initial 15 sec of PORH. As a result, the BFI and MVC peaked at the same level as in the control condition, but in less time. It is noteworthy, however, that the BFI and MVC returned toward resting levels after the peaks more quickly, as evidenced by the significant decreases in the 50% decay times. Consequently, the total vasodilation during the first 1 min and 5 min of PORH were diminished. Thus, in the face of potent local vasodilatory stimulation, sympathoexcitation still effectively constrained vasodilation within the skeletal muscle microvasculature by decreasing its duration. At the same time, the sympathoexcitation significantly reduced the peak vasodilation in the skin microcirculation. Simultaneous quantification of the BFI and LDF on a second‐to‐second basis using DCS and laser‐Doppler flowmetry revealed, for the first time, the different effects of the integration of sympathetic vasoconstriction and local vasodilation in the human skeletal muscle and skin microcirculation.

The sympathetic inhibition of the local vasodilation in both skeletal muscle and skin microvasculature were blunted by lengthening of the occlusion period. In addition, prolonging the ischemia caused greater blunting of the inhibitory sympathetic effect on initial and peak vasodilatory responses in skeletal muscle than skin. The greater and more prolonged tissue hypoxemia and increased production of vasodilatory substances due to the longer ischemia may contribute to blunting the adrenergic vasoconstriction (Carlsson et al. 1987; Thomas et al. 1994; Tschakovsky et al. 2002; Kaur et al. 2015; Hearon et al. 2017). Moreover, because skeletal muscle has a higher metabolic rate than skin (Fewings et al. 1970; Yu et al. 2005), more vasodilating metabolites would be expected to accumulate in skeletal muscle during ischemia and induce greater blunting of the sympathetic inhibition of vasodilation. Alternatively, because decreases in intravascular pressure evoke endothelial derived‐hyperpolarization (EDH) (Bagher et al. 2012), which opposes sympathetic vasoconstriction (Kurjiaka and Segal 1995; Hearon et al. 2016), myogenic vasodilation may also be involved. It has been shown in isolated rat arteries (Jasperse et al. 2015) and arterioles (Koller and Bagi 2002) lacking parenchymal tissue that the magnitude of reactive dilation in response to intravascular pressure reduction depends on the duration of the pressure reduction. Therefore, after more prolonged occlusion, the myogenic vasodilation may be enhanced through mechanisms within the vascular wall, such as EDH, and not from metabolic factors released from parenchymal tissue. This may result in a more effective inhibition of sympathetic vasoconstriction.

The acceleration of the initial vasodilation by sympathoexcitation, which occurred selectively in the skeletal muscle microvasculature during PORH after the 10‐min occlusion, is an intriguing phenomenon. One of its effects is to prioritize blood flow to the skeletal muscle over flow to the skin. Our finding indicates that against a background of robust vasodilatory stimulation, sympathoexcitation enhances or speeds the mechanism(s) that induce the initial peak vasodilation in the skeletal muscle microcirculation. The peak and duration of PORH in the forearm appears to be determined by different vasodilatory substances and signaling pathways, though with some redundancy (Carlsson et al. 1987; Engelke et al. 1996; Crecelius et al. 2013). For example, inhibition of K_IR_ channels reduces both the peak and duration of hyperemia (Crecelius et al. 2013), while inhibition of ATP‐sensitive potassium channels (Banitt et al. 1996) or Na+/K+‐ATPase (Crecelius et al. 2013) reduces the duration of the hyperemia but not the peak response. Several studies have shown that nitric oxide makes only a modest contribution, if any, to peak reactive hyperemia, but it does contribute to the duration of the response (Dakak et al. 1988; Tagawa et al. 1994; Meredith et al. 1996). On the contrary, prostaglandins largely contribute only to determining the peak reactive hyperemia (Engelke et al. 1996; Addor et al. 2008), or to both to peak and duration of PORH (Carlsson et al. 1987). It is unlikely, however, that the sympathoexcitation induced by a short period of forehead cooling was sufficient to modify production of vasodilator autacoids in occluded skeletal muscle.

Alternatively, the enhanced initial vasodilatory response may be associated with myogenic mechanisms. Notably, Jasperse et al. (2015) showed that reactive dilation of isolated rat soleus feed arteries upon restoration of intravascular pressure after its earlier reduction is greater when the intravascular pressure recovered to a higher baseline pressure than when it recovered to a lower baseline pressure. Similarly, they also showed in the human forearm that peak PORH is greater when intravascular pressure is increased by setting the arm below the heart than when it is reduced by setting the arm above the heart. Given these findings, the myogenic vasodilation observed in this study may be enhanced under sympathoexcitation, as skeletal muscle microvessels were reperfused at an elevated arterial pressure. However, this response may only be manifest in the context of potent vasodilatory stimulation that blunts sympathetic vasoconstriction. This is because the initial vasodilation was not enhanced by a similar increase in arterial blood pressure under sympathoexcitation in the 70‐sec occlusion trials.

Compared to PORH in the forearm, PORH in the skin is smaller in magnitude with a markedly delayed peak, but is significantly longer in duration (Sinoway et al. 1988; Moradkhan et al. 2007; Addor et al. 2008). The differences in these responses reflect, in part, the different characteristics of PORH in the skeletal muscle and skin microvasculature. Our results are consistent with those earlier studies and also provide direct evidence that PORH in the human skeletal muscle microvasculature has significantly different properties than that in the skin microvasculature. In both the 70‐sec and 10‐min occlusion trials, peak PORH in skin was lower than in skeletal muscle. In the 70‐sec occlusion trials, the time to peak hyperemia in the skin was similar to that in skeletal muscle, but the hyperemia duration was slightly shorter. In the 10‐min occlusion trial, by contrast, the time to peak and duration of the hyperemia were significantly longer in skin than skeletal muscle. The delayed peak and extended duration of the hyperemia in skin compared to skeletal muscle was only observed in PORH after sufficiently long occlusion periods. The higher PORH peak in skeletal muscle than skin may be due to a greater accumulation of vasodilatory metabolites during ischemia in skeletal muscle than skin (Fewings et al. 1970; Yu et al. 2005). However, the longer hyperemia duration and the greater total PORH during the 5‐min period after cuff release in skin in the 10‐min occlusion trials cannot be explained by a lower vasodilatory metabolite concentration in skin than skeletal muscle. It suggests the different characteristics of PORH in the two tissues reflect differences in the intrinsic nature of the microvessels. Our results agree with an earlier study showing that the rate of rise to peak reactive hyperemia in skin and the rate of response decay both decrease with increases in the duration of occlusion (Wilkin 1987). It was suggested that the dependence of the decreases in these two rates on the lengthening of ischemia is consistent with the viscoelastic characteristics of resistance vessels rather than changes in the concentration of vasodilator substances produced during occlusion (Wilkin 1987). It may be that differences in the viscoelasticity of microvessels in skeletal muscle and skin contribute to the different characteristics of PORH in the two tissues. Alternatively, it is possible that hyperemia‐induced warming of the skin produces vasodilation that prolongs the duration of hyperemia.

Two earlier studies examined the simultaneous effects of sympathetic vascular tone on PORH in the forearm and skin by measuring brachial artery blood flow and LDF (Sinoway et al. 1988; Moradkhan et al. 2007). Sinoway et al. (1988) showed in five subjects that sympathoexcitation induced by forehead cooling during PORH after occlusion for 10 min significantly decreases maximum vasodilation in the forearm but has no significant effect on peak skin hyperemia. In addition, Moradkhan et al. (2007) demonstrated in twelve subjects that suppressing regional sympathetic activity in the forearm using a Bier block with phentolamine after occlusion for 10 min substantially elevates forearm hyperemia and also tends to enhance skin hyperemia (*P* = 0.07). These different observations in forearm and skin may be due, in part, to differences in the modulation of PORH in skeletal muscle and skin by sympathetic vascular tone. However, because brachial artery blood flow includes blood flow to both skeletal muscle, skin and other tissues, the differences in the responses between the skeletal muscle and skin are not revealed by these results. We provide direct evidence of differential modulation of local vasodilation by sympathetic nerves in the skeletal muscle and skin microvasculature. Our results in a relatively large number of subjects (*n* = 36 for LDF measurements) confirmed the results of Sinoway et al. (1988), who showed that sympathoexcitation does not significantly reduce peak PORH in skin after a 10‐min occlusion. We also found that peak skin vascular conductance, which was not reported in the earlier study, was significantly reduced by the sympathoexcitation. To our knowledge, this is the first study investigating the effects of sympathoexcitation on PORH in skin induced by occlusions of different length. The sympathetic inhibition of PORH in skin was less clear in the 10‐min than 70‐sec occlusion trials. Indeed, despite the significant reduction in peak vasodilation by sympathoexcitation in the 10 min occlusion trials, the duration of vasodilation and the total vasodilatory response did not significantly decrease. This is clearly in contrast to the finding in skeletal muscle that sympathoexcitation in the 10‐min occlusion trials had no inhibitory effect on peak vasodilation but significantly reduced the duration of vasodilation and total vasodilation. Therefore, the effects of sympathoexcitation on PORH in skeletal muscle and skin depend on the length of ischemia and also whether one is considering the peak or the duration of the response.

### Limitations and methodological considerations

Because photons emitted from the DCS probe pass through the overlying tissues before reaching the skeletal muscle (e.g., skin and adipose tissues), the detected diffusion characteristics may be affected by these overlying tissues. However, it has been demonstrated that signals detected with a large interprobe distance (>2 cm) derive predominately from the muscle layer when the thickness of the near‐surface layers is small (~5.5 mm) (Yu et al. 2005). Our experimental setting (i.e., interprobe distance = 3 cm; thickness of near‐surface layers at the DCS measurement site = 3.0 ± 0.2 mm) satisfy those criteria. Furthermore, in this study, we demonstrated that responses in BFI and LDF during PORH after the 10‐min occlusion significantly differed in both magnitude and duration. It should be noted that LDF continued to increase while BFI was decreasing after the peak. Indeed, the time to peak of LDF was closer to the 50% decay time of BFI than the time to peak of BFI. These results provide new evidence that BFI derived using a large interprobe distance provides different blood flow information than the LDF. Considering all these findings, we believe that the observed BFI responses mainly reflect the blood flow responses in the skeletal muscle microvasculature.

In this study, the PORH responses of skeletal muscle and skin could not be evaluated in absolute values, due to technical limitations of DCS and laser‐Doppler flowmetry. Because BFI and LDF were assessed as relative changes from those resting levels, the quantitative PORH responses (e.g., PORH peak) were influenced by the resting values. Therefore, when comparing quantitative PORH responses in absolute values between skeletal muscle and skin, it may be different from observations in this study. However, the time responses (e.g., time to peak) and qualitative changes (e.g., the initial vasodilation was accelerated only in skeletal muscle) of PORH would not be influenced by the resting levels. The observed differences in time responses and qualitative changes of PORH between skeletal muscle and skin support our conclusion that the integration of sympathetic vasoconstriction and local vasodilation has different effects in the skeletal muscle and skin microvasculature.

We performed forehead cooling, which is a well‐established method for evoking sympathoexcitation, peripheral vasoconstriction and increases in arterial blood pressure (Sinoway et al. 1988; Heindl et al. 2004; Muller et al. 2014; Ichinose et al. 2018). The observed mild bradycardia during forehead cooling would be due to rapid enhancement of cardiac vagal tone elicited through stimulation of trigeminal nerve endings (Heindl et al. 2004). There may be a difference in the degree of activation of sympathetic nerves innervating the skeletal muscle and the skin during forehead cooling. In the 70‐sec occlusion trial, forehead cooling tended to reduce the peak and total vasodilation responses during PORH more in skeletal muscle than skin. In the 10‐min occlusion trials, however, forehead cooling reduced peak vasodilation only in the skin, while initial vasodilation was selectively accelerated in the skeletal muscle. It is difficult to explain these results based solely on a difference in the degree of sympathetic nerve activation in skeletal muscle and skin. Our results more likely reflect differences in the integration of sympathetic vasoconstriction and local vasodilation in these tissues. Future studies, including measurements of sympathetic nerve activity, will be needed to shed additional light on differences in microvascular regulation in skeletal muscle and skin.

If forehead cooling decreased forearm skin temperature, skin vascular responses during PORH could be affected, since skin vascular regulation is largely temperature dependent. The forehead cooling is a cold stimulus restricted to the forehead and does not directly decrease forearm skin temperature, though forearm skin temperature may decrease if forehead cooling decreases forearm skin blood flow. In this study, forehead cooling began during occlusions. Thus, forearm blood flow was similar (i.e., completely occluded) in both the control and forehead cooling conditions before release of the occlusion. During PORH, although forehead cooling decreased peak skin vascular conductance, peak skin blood flow did not significantly change. In addition, although forehead cooling decreased total perfusion in skin during the first min in the 70‐sec occlusion trials, it had limited effect on total skin perfusion during the first 5 min in both the 70‐sec and 10‐min occlusion trials. These results make it unlikely that forearm skin temperature was meaningfully affected by forehead cooling over the entire 5‐min period of PORH.

In this study, most of the subjects were male (*n* = 37), though two females were also studied. Consequently, our conclusions are based primarily on data from young males. Although the small number of females precluded making a reliable statistical comparison between the responses of female and male subjects, we did not see any important gender differences in the results. Nevertheless, additional studies are needed to further examine these responses in females and to test for gender differences in responses.

In summary, by simultaneously employing DCS and laser‐Doppler flowmetry, we found that in humans, integration of sympathetic vasoconstriction and local vasodilation has different effects on the skeletal muscle and skin microvasculature. This difference in the integrated effects of two vascular control mechanisms would be importantly involved in selective control of perfusion in the microcirculations of different tissues.

## Conflict of Interest

Authors have no competing interests to declare.
